# Rest in the Treatment of Bright’s Disease

**Published:** 1908-04

**Authors:** H. F. Harris

**Affiliations:** Atlanta, Ga.


### ORIGINAL COMMUNICATIONS.

---

### REST IN THE TREATMENT OF BRIGHT'S DISEASE.

BY H. F. HARRIS, M. D., ATLANTA, GA.

The therapeutic value of rest must have been more or less appreciated even before the dawn of the medicine of to-day, but it was not until John Hilton wrote his masterly monograph on this subject that the especial attention of the medical profession was turned in this direction. Even Hilton, however, regarded the matter rather from a surgical than a medical aspect, and though the credit is due him that he first distinctly recognized and directed attention to this most powerful of therapeutic agencies, it is clear that he did not appreciate its value in the treatment of diseases of the internal organs. Somewhat later Weir Mitchell introduced rest in the practice of medicine as distinguished from surgery, and as the result of his advocacy this agency is now universally recognized as the most potent in correcting the curious nervous states that result from over work, or, what is much more frequently the case, from the various forms of indigestion. Notwithstanding the great degree of merited favor which the Mitchell plan of treatment has attained, it appears to me that the profession as a whole has not as yet appreciated the wonderful potency of rest in the treatment of disease-conditions generally. If I be correct in this view there is no question that we discard assistance of the most powerful kind in our combat with disease.

The experience of recent years has convinced me that one of the great faults in the treatment of that group of conditions known as Bright's disease has been the almost total disregard of the necessity of proper rest. This failure on our part to apply well known principles in this particular case has probably in a measure resulted from a rather indefinite idea as to the causation of the various kinds of chronic inflammation of the kidneys. We have been disposed to look upon these conditions being for the most part inevitable, and a consequence of mysterious agencies, the exact nature of which are entirely unknown. This feeling has been undoubtedly strengthened by the notorious failure of all ordinary therapeutic agencies in the treatment of this disease. It is true that we have long recognized that when patients are placed on milk—the best and most easily assimilable of all foods—they more or less improve, but even where this plan of diatetic treatment could be rigidly carried out, we have expected the patient gradually to go from bad to worse and ultimately die. The object of this paper is to suggest the combination of proper dieting with rest,—this, when properly carried out, appearing to me to promise more than any other method of treatment.

In order that we may at least have a theoretical understanding of the why and wherefore of the above suggestions, a brief consideration of the causation of Bright's disease may not be without interest.

It has long been recognized that during the course of any of the ordinary acute infectious diseases inflammations of the kidneys may supervene—in most cases quickly subsiding, but in others terminating finally in the chronic disease—conditions that we call Bright's; apparently the latter are of frequent occurrence. It should also be remembered that in some of those instances where Bright's apparently results from acute diseases, the patients have previously suffered from an unrecognized chronic inflammation of the kidneys, which are only diagnosticated after having been made worse by the acute malady in question. In another class of cases we have long looked upon alcohol, lead poisoning, and gouty states as being etiologic features, but in this country, at least, it is only occasionally true that we can trace antecedents of this kind for instances of chronic Bright's. To what then shall we ascribe the production of these cases of Bright's that are clearly not the result of the supposed usual causes?

I am naturally averse to theorizing, and look upon with the greatest suspicion all conclusions established by this very questionable method of reasoning.

However, where facts seem to more and more square with our theoretical deductions as we look into a matter more closely, the time is finally reached when we may be authorized in putting forward tentative views, and such is my attitude in this instance. For many years it has seemed to me in the highest degree probable that almost all cases of Bright's disease are due to chronic "indigestion." I am unfortunately not in a position to absolutely specify the precise nature of the particular kind of imperfect digestion that I think gives rise to these inflammations. Indeed there can be no question that at the present time we possess no very accurate knowledge of the exact character of the different states that are quite vaguely known as "indigestion," and I believe that we can only hope for a better understanding of this subject from a patient chemical and bacteriological investigation of the intestinal excretions in these conditions. Certain assumptions, however, appear to me as being highly probable. Bright's disease seems much more common and the affection comes on much earlier in life in those persons who are of a corpulent tendency, and particularly where such individuals are rather free-livers. The comparative immunity of thin persons, it seems to me, may be a consequence of the fact that they are usually more or less dyspeptic, and that they generally find that overeating makes them much worse. I do not believe alcohol is very often directly responsible for Bright's, but I have frequently seen it occur in people who make a habit of taking a drink or two before meal times, or in those who drink wines or beer with their food. In both of these cases the alcohol probably acts in the way of producing an excessive and unnatural appetite, and, as a consequence, more or less indigestion occurs. It has long been recognized that persons who drink very excessively do not have gout, and my own observations point to the probability that such individuals rarely have Bright's. The probable explanation is that unfortunates of this kind eat very little, and, as a consequence, do not have the chronic forms of indigestion occasioned by the excessive taking of food. Much of the clinical evidence in support of the view that Bright's results from some form of chronic indigestion is obtained from the urine. An examination of this excretion in the earlier stages of the affection will usually show it to contain, in addition to albumen and casts, an increase in indican and skatol, and the Ehrlich paradimethyamidobenzaldehyde reaction is most pronounced. On microscopic examination we often find oxalates, and not infrequently uric acid.

If we now accept the suggested causation of Bright's, the first obvious deduction would be that patients should be placed on some diet that would, while being sufficient for all of the needs of the organism, be easily digested and assimilated. For this food we would naturally turn to milk, which, as we all know, has been regarded universally as the most efficient method of treating this terrible disease. To this accepted method of treatment, I would add rest, for what seems to me to be the following good reasons:

It is well established that a person at rest needs practically one-half of the amount of nourishment required while at active work, and we, of course, know that the functional activity of the kidneys depends upon the amount of chemical change that goes on in the body. It is not necessary to argue to the point that the diseased organ should have as much rest as possible, and if we can accomplish this by putting the patient at rest, limiting the amount of food and thus decreasing the work that the kidneys have to perform, these organs would be placed in the best possible condition for gradual restitution. Of course, I would not assume for a moment that a treatment of this kind could restore kidney tubules that are entirely destroyed, but the fact that the patient is living is evidence that those still remaining are competent to keep life going, and if we can put a stop to the further advance of the disease, the patient should be able to live out his allotted years.

Unfortunately this treatment requires considerable time, which makes the collection of statistics an exceedingly slow matter. I have seen a patient who had undoubtedly suffered from Bright's for a number of years previously apparently entirely recover in eighteen months. In this case the urine was loaded with casts and albumen, and the patient just prior to the inauguration of the treatment had suffered two attacks of apoplexy, and a few months later had had a hemorrhage into the retina of one eye. I have another patient who has been on this treatment for about seven years, and although he had lost complete sight of both eyes at the time he was first seen by me, he is apparently now well in every other particular. For a number of years now he has not adhered strictly to the treatment. I have a number of cases at present under observation who have shown most pronounced good effects as a consequence of the treatment. and it seems to me that if it could be carried out thoroughly that our results would be infinitely better than they are at present. At this point I would say that under rest in bed and the milk diet the urine of patients frequently becomes normal in two or three months, but if they begin to take exercise or resume ordinary foods the albumen quickly reappears; the treatment should, therefore, last over a period of at least a year, and perhaps longer in most instances. When the patient begins to resume his ordinary method of living the urine should be frequently examined, and if evidence of trouble reappears the patient must again be placed on the treatment.

I believe that the rest treatment should be taken under conditions where the patient can get the maximum amount of fresh air. He should, of course, at all times be warmly clad.

I believe that a small dose of morphia daily materially assists in limiting tissue change, and can usually be given with advantage.

It is very important that the patient's bowels be kept open.

This communication is in the nature of merely a preliminary paper; at some future time I hope to give to this Association a statistical report of the results achieved.

